# Neck and Shoulder Pain with Scapular Dyskinesis in Computer Office Workers

**DOI:** 10.3390/medicina59122159

**Published:** 2023-12-13

**Authors:** Seong Eun Moon, Young Kyun Kim

**Affiliations:** Graduate School of Sports Medicine, CHA University, Seongnam 13496, Republic of Korea; daiske106@naver.com

**Keywords:** computer office workers, scapular dyskinesis, neck pain, shoulder pain

## Abstract

*Background and Objectives:* Computer office workers spend long periods in front of a computer, and neck and shoulder pain are common. Scapular dyskinesis (SD) is associated with neck and shoulder pain. However, SD in computer office workers has not been elucidated. We aimed to investigate the prevalence of SD, neck and shoulder pain, disability, and working hours in computer office workers. *Materials and Methods:* In total, 109 computer office workers participated in this study. The results of a scapular dyskinesis test (SDT), lateral scapular slide test (LSST), neck disability index (NDI), shoulder pain and disability index (SPADI), visual analog scale (VAS) scores of the neck and shoulder, and working hours were recorded. *Results:* Ninety-eight computer office workers (89.9%) had SD. Computer office workers with SD had significantly higher NDI (*p* = 0.019), neck VAS (*p* = 0.041), and dominant shoulder VAS scores (*p* = 0.043). The LSST results showed a significantly greater distance (*p* = 0.016) in participants with SD. *Conclusions:* The prevalence of SD was very high in computer office workers, and neck and shoulder pain were more prevalent in workers with obvious SD.

## 1. Introduction

Computer office workers spend a great amount of time in front of computers, and neck and shoulder problems have increased as a result [1]. Musculoskeletal pain has become common among computer office workers with poor workstation ergonomics [2]. Using a keyboard for 4–6 h per day at work increases the risk of neck and shoulder pain four times compared to minimal users [3], and computer office workers spend considerable time on computers and experience neck pain [4]. According to Silvian et al. and Kashif et al., computer office workers report that neck pain is the most common musculoskeletal disorder (MSD) [5,6]. Computer office workers tend to work in a forward head posture for prolonged hours [5], which may cause significant stress to the supporting soft tissues of the cervical spine and may affect scapular and shoulder function, causing altered scapular kinematics [7,8], defined as scapular dyskinesis (SD) [9].

SD is not an injury but is related to shoulder injuries [10]. However, studies have recently reported that neck pain is also related to SD [11,12,13]. Compared to the healthy population, those with neck pain show altered scapular position and/or movement [11,14]. Proper alignment and function of the scapula are very important because the link between the scapula and humerus must work efficiently to deliver force [10]. SD can create mechanical dysfunction between the neck and scapula, which share muscle attachments, possibly causing recurrent neck pain [15]. SD can also contribute to neck and shoulder pain and increase the risk of shoulder pain by 43% [16,17]. Therefore, those who spend long periods working on a computer might be at risk of developing SD, which can lead to neck and shoulder pain.

The glenohumeral joint is inherently unstable; therefore, scapular and humeral stability rely heavily on the soft tissue of the shoulder [18]. The scapula acts as a bridge between the cervical spine and the shoulder, providing mobility and stability to the neck and shoulder [19]. Therefore, dynamic stability of the scapula relies on the periscapular muscles [20]. The scapular dyskinesis test (SDT) is used to identify SD, which includes dynamic shoulder movement [21]. The lateral scapular slide test (LSST) is another test for SD which measures the distance between the thoracic spine and the scapular inferior angle [22]. Previous studies have applied the LSST to investigate SD [8,23,24,25]. However, the test only measures the distance between the thoracic spine and the inferior angle of the scapula in three positions. Moreover, the contralateral side ise assumed to be normal with LSST. Therefore, it may not be suitable for detecting SD [26]. The LSST is suitable to measure the static posture of the scapula but does not include dynamic movement measurements [26,27]. As scapular motion occurs in a three-dimensional pattern with shoulder movement [21], a dynamic shoulder motion is recommended to identify SD rather than static position measurements [28,29]. Therefore, the SDT is recommended to measure SD with dynamic shoulder motion [28,30].

In total, 71% of computer office workers spend more than 8 h per day at work [31]. Computer office prolonged work hours increases the risk of musculoskeletal pain [32], and more than 8 h of work per day increases the risk of neck and shoulder pain [33]. Furthermore, neck and shoulder pain are the most common musculoskeletal pains among office workers [24,34]. SD can decrease neck and shoulder stability [19], and a high incidence of neck and shoulder pain may be related to SD for computer office workers. However, investigations of the prevalence of dynamic and static SD and neck and shoulder pain in computer office workers are lacking. The scapula kinematics with SD in shoulder pain patients were varied [16]. Ozunlu et al. reported there was no relationship between shoulder pain and SD [26]. However, Cools et al. reported shoulder pain and SD are linked [19]. Therefore, further research is necessary to investigate shoulder pain and SD. We, therefore, aimed to investigate the prevalence of SD in computer office workers and the related neck and shoulder pain and disability. We hypothesized that the prevalence of SD among computer office workers would be higher than previously reported, and that neck and shoulder pain and disability will increase as SD worsens. The primary objective of the study was to compare neck and shoulder pain and disability, and the distance between the thoracic spine and scapula according to the severity of SD. The secondary objective was to compare the severity of SD according to neck and shoulder pain and disability.

## 2. Materials and Methods

### 2.1. Instructions

This study adhered to the STROBE Guidelines during the search and reporting phases of the research process. The STROBE Guidelines include a 22-item checklist to clearly present what is planned and conducted in an observational study [35].

### 2.2. Participants

This cross-sectional, single-blind study was conducted at the Graduate School of Sports Medicine, CHA University, Seongnam. Participants were recruited from information technology (IT) companies in Seoul via email from 12 April to 11 September 2023. The inclusion criteria were software designers, developers, coders, or any computer office workers working at least 4 h a day, 40 h per week; with more than 1 year of work experience; and aged 20 to 50 years [36]. We excluded workers with acute neck or shoulder injuries within 1 month; neck or shoulder surgery, fracture, or traumatic injuries; and an inability to perform SDT and LSST [17].

The sample size was calculated based on a previous study reporting an SD prevalence of 41.7% among office workers [17,24]. We used the expected proportion (p) of 0.42, and the precision (d) was set at 0.1. A prior sample size calculation was performed (G*Power software 3.1) using the expected proportion (p) that was calculated for SDT of 0.42 [24] (5% error level). Considering a 5% dropout rate, 109 participants were recruited. The Institutional Review Board of CHA University reviewed the study (1044308-202303-HR-070-02) and written informed consent was obtained prior to measurements.

### 2.3. Protocol

**Figure 1:**
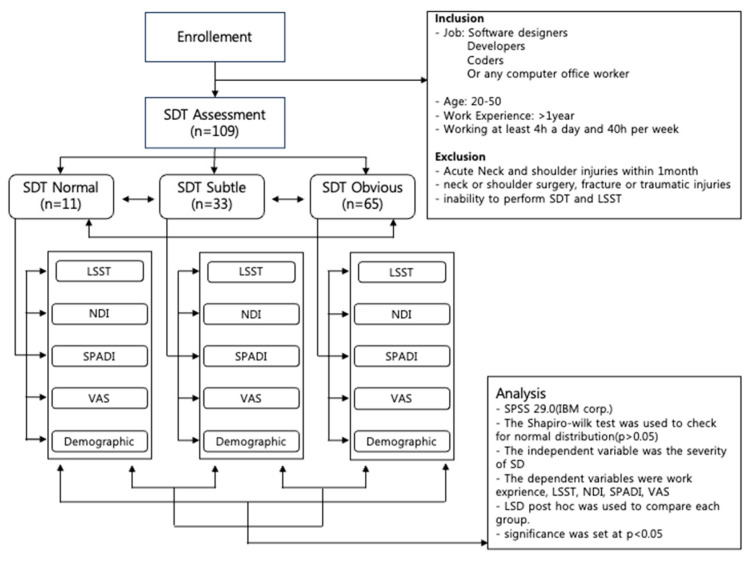
Flowchart of measurements. SDT: Scapular dyskinesis Test; LSST: Lateral Scapular Slide Test; NDI: Neck Disability Index; SPADI: Shoulder Pain And Disability Index; VAS: Visual Analogue Scale.

All participants were asked to provide their age and work experience (total years of work experience, working hours per day, week, month, and rest hours per day) [31]. Then, the SDT was used to measure SD, and LSST was used to measure the distance between the thoracic spine and scapula. The investigator for SDT and LSST was a musculoskeletal rehabilitation trainer with 8 years of experience in the field and 25 years of clinical experience as a certified athletic trainer. We calculated our intra-reliability with SDT and LSST by repeatedly measuring 10 participants (SDT k = 0.78; LSST k = 0.76). Neck disability index (NDI), shoulder pain and disability index (SPADI), and neck and shoulder visual analog scale (VAS) results were recorded. According to the SDT results, all data were compared to investigate the differences. The investigator for SDT was YK, and SM measured LSST. The results were blinded to each other. The participants were also blinded to the results of SDT and LSST while VAS, NDI, and SPADI were measured (Figure 1).

### 2.4. SDT

Participants were asked to grasp dumbbells according to their body weight as follows: <68.1 kg participants received 1.4 kg dumbbells, and >68.1 kg received 2.3 kg dumbbells. The participants were asked to flex their shoulders to 180° and return to a neutral position with their thumbs up, at a rate of 3 s flexion and extension. This process was repeated five times to analyze SD [21]. The palpation method was used to perform the SDT [27]. The 3 grade SDT was applied to test the severity of SD [27]. Scapular movement was considered normal when there was no evidence of abnormalities. Mild abnormal movements were recorded as subtle; apparent abnormal movements were recorded as an obvious abnormality [21]. The reliability of the SDT was moderate to substantial (k = 0.48–0.64) [21,27].

### 2.5. LSST

Participants were asked to stand, and three different positions were used to measure the distance between the thoracic spinous process of T7 and the inferior angle of the scapula using a caliper (CAS, Yangju city, Gyeonggi-do, Republic of Korea) with arms on the sides, hands on the hips, and arms at 90° abduction with internal shoulder rotation [22]. Each measurement was performed twice, and the mean value was calculated for the data analysis. A difference of ≥1.5 cm bilaterally was considered positive for SD [26]. However, we applied LSST, but only as a measure of the distance between the scapula and thoracic spine. The reliability of the LSST has been reported to be good (ICC > 0.87) [37].

### 2.6. NDI

Participants were asked to complete the NDI questionnaire, the most widely used questionnaire to assess neck pain and disability [38]. We used the Korean version, and the reliability of the NDI was good (ICC = 0.93) [39]. The questionnaire is scored out of a possible 100 points, with a higher score indicating worse neck pain and disability [40]. NDI reported a Minimal Clinically Important Change (MCIC) of 3.5 points using ROC curve, and a Minimal Detectable Change (MDC) of 10.5 points [41].

### 2.7. SPADI

Participants were asked to complete the SPADI to measure shoulder pain and disability, and dominant and non-dominant scores were measured separately. The scores ranged from 0 to 100, with higher scores indicating greater shoulder disability [42]. SPADI reported a MCIC of 8 points [43], and a MDC of 18 points [44]. The reliability of the SPADI was high (ICC = 0.85) [45].

### 2.8. VAS

A VAS was used to measure the worst neck and shoulder pain felt by the participant in one week. A 100-mm horizontal line was used for the VAS, and participants were asked to rate their pain from a minimum of 0 to a maximum of 10 points [46].

### 2.9. Statistical Analysis

SPSS version 29.0 (IBM Corp. SPSS Inc., Chicago, IL, USA) was used for statistical analysis. The parametric statistical method, a one-way analysis of variance (ANOVA), was used to analyze the data. The Shapiro–Wilk test was used to check for normal distribution. The overall data examined using the Shapiro–Wilk test was observed to be normal distribution (*p* > 0.05). The independent variable was the severity of SD, and the dependent variables were work experience, LSST, NDI, SPADI, and VAS. LSD post hoc test was used to compare each group. The level of significance was set at *p* < 0.05.

## 3. Results

A total of 109 computer office workers participated in the study; most participants were adults in their 30 s with more than four years of experience and working at least eight hours a day. There were no significant differences in age (*p* = 0.236); sex (*p* = 0.787); total IT work experience (*p* = 0.906); working hours per day (*p* = 0.897), week (*p* = 0.824), or month (*p* = 0.397); or break hours per day (*p* = 0.261) (Table 1) between those participants with and without SD.

A total of 109 computer office workers participated in the study. Of the 109 computer office workers who participated in the experiment, 98 (89.9%) had SD, 65 (59.63%) showed obvious SD, and 33 (30.28%) presented with subtle SD. In the dominant arm, 51 participants (46.79%) had obvious SD, 42 (38.53%) had subtle SD, and 16 (14.68%) had normal SD. In the non-dominant arm, 44 (40.37%) participants showed obvious SD, 40 (36.70%) showed subtle SD, and 25 (22.94%) showed normal SD (Table 2).

A total of 104 (95.4%) of the 109 computer office workers reported neck pain. Sixty-four (61.54%) participants with neck pain showed obvious SD, 31 (29.81%) had subtle SD, and nine (8.65%) were normal. Five participants reported no neck pain, one had obvious SD, two had subtle SD, and two were normal. Ninety participants (82.5%) reported neck and shoulder pain. Among them, 56 (62.92%) showed obvious SD, 27 (30.0%) showed subtle SD, and seven (7.78%) were normal. Pain in the dominant arm was observed in 23 (21.1%) participants; 14 (60.87%) showed obvious SD, seven (30.34%) showed subtle SD, and two (8.7%) were normal. There were three (2.8%) participants with pain in the non-dominant arm; one (33.43%) had subtle SD, and two (66.67%) were normal. Of the 17 (15.6%) participants with no shoulder pain, nine (62.22%) had obvious SD, five (29.41%) had subtle SD, and three (17.65%) had normal SD, respectively (Table 3).

There were significantly different NDI scores (*p* = 0.019) among the normal (5.82 ± 3.05), subtle (11.76 ± 2.54), and obvious (10.14 ± 1.36) SD groups; however, there was no significant difference between the subtle and obvious group. There were significantly different neck VAS scores (*p* = 0.041) among normal (2.4 ± 1.09), subtle (3.92 ± 0.67), and obvious (3.61 ± 0.41) SD; however, there was no significant difference between the subtle and obvious group. There were no significantly different SPADI scores (*p* = 0.243) in dominant shoulders among the three different SD groups; however, there was a tendency for the normal (4.91 ± 4.67) group to show lower SPADI scores compared to the subtle (11.39 ± 3.85) and obvious (10.72 ± 3.01) groups. There was no significant difference in SPADI scores with the non-dominant shoulder (*p* = 0.267); however, there was a tendency for the normal group (2.45 ± 2.07) to show lower SPADI scores than the subtle (7.58 ± 3.42) and obvious (7.06 ± 2.42) groups. There were significantly different VAS scores with the dominant shoulder (*p* = 0.043) (normal, 1.52 ± 1.22; subtle, 3.21 ± 0.81; obvious, 2.85 ± 0.42); however, no significance (*p* = 0.257) was found with the non-dominant arm (Table 4).

There was a significant difference (*p* = 0.017) in the distance from the LSST 2 position, whereby the distances in the normal group (2.88 ± 1.31 cm) were shorter than in the subtle (6.1 ± 1.49 cm) and obvious (7.38 ± 1.34 cm) groups. No significant difference was observed, but a tendency in the LSST 1 (*p* = 0.135) and LSST 3 positions (*p* = 0.053) was observed (Table 5).

## 4. Discussion

Ninety-eight computer office workers (89.9%) had SD. Computer office workers with SD had significantly higher NDI, neck VAS, and dominant shoulder VAS scores. The LSST results showed a significantly greater distance in those with SD. We identified an SD prevalence of 89.9% (98/109) in computer office workers. Previous studies have reported 90% (90/99) [17], 41.7% (15/36) [24], and 34.1% (28/81) SD prevalence [47]. Our results were very high, similar to those of Vongsirinavarat et al. in which SDT was utilized [17]. However, LSST applied in two other studies showed a very low SD prevalence [24,47]; overhead athletes accounted for 61% of the study population [48], which was much lower than in our results. Overhead athletes can exhibit SD due to repetitive overhead throwing using the scapular thoracic muscles [49,50]. Office workers experience prolonged sitting, poor work ergonomics, poor posture, repetitive work, neck and shoulder muscle fatigue, and pain [25]. This may lead to altered periscapular muscle activation, and SD occurs [30]. Recent studies on athletes have reported an SD prevalence of 52.7% in elite boxers and 64.4% in Brazilian jiu-jit-su (BJJ) athletes [13,51]. Boxers experience repetitive punching, and being punched in the face could lead to SD due to neck pain. BJJ athletes with repetitive grappling techniques could experience SD in their shoulders [13,51]. Computer office workers show a very high prevalence of SD, and there must be different risk factors causing this phenomenon compared to athletes, such as neck and shoulder pain.

The prevalence of neck pain was 95.4% (104/109) among computer office workers, and 91.3% (95/104) showed SD. Although there were only five participants without neck pain, 60% (3/5) showed SD and 40% (2/5) were not in the no neck pain group. Moreover, the neck pain group had 61.54% (64/104) participants with obvious SD, and 20% (1/5) of the obvious SD participants were present in the no neck pain group. Therefore, computer office workers with neck pain have a higher incidence rate of SD and more severe SD than those without neck pain. Neck pain with SD results in decreased activation of the middle trapezius muscle, and alteration of the scapulothoracic muscles could lead to neck pain [15]. A previous study also reported that a chronic neck pain group showed a higher SD prevalence rate than a no neck pain group [52]. According to Thigpen et al., forward head and round shoulder posture individuals without shoulder pain have altered scapular motion [53]. SD in computer office workers is related to neck pain. Moreover, shoulder with pain computer office workers showed a high prevalence of SD in the study (91.3% with dominant shoulder pain) (Table. 3). However, only a few participants reported no neck or shoulder pain. Therefore, further investigation is necessary. SD is directly related to shoulder pain and injury and contributes to shoulder pain [10]. Therefore, such a high incidence rate of SD in computer office workers might be related to both neck and shoulder pain.

Computer office workers with subtle and obvious SD had significantly higher NDI and neck VAS scores than those with normal SD. Depreli et al. report a lower average NDI score in computer office workers of 4.78 ± 3.2 [24] compared to our results. The MCIC in NDI using a receiver operating characteristic (ROC) curve was 3.5 points [41], with an MCIC observed between the normal and SD groups, but no MCIC observed between the subtle and obvious groups. In addition, the MDC of the NDI was 10.5, and no MDC was observed between the normal and SD groups. Therefore, the difference in pain between the normal and SD groups may be due to the margin of error of the questionnaire measurement [41] (normal, 5.82 ± 3.05; subtle SD, 11.76 ± 2.54; obvious SD, 10.14 ± 1.36). An NDI score of 0–4 indicates no disability, 5–14 indicates mild disability, and 15–24 indicates moderate disability [54]. Depreli et al. [24] reported no neck disability, whereas our study reported mild disability. Additionally, 36 computer office workers participated in the previous study, whereas 109 workers participated in our study. The average neck VAS score was 2.5 ± 3.33 in the previous study, whereas our results showed 2.4 ± 1.09 (normal SD), 3.92 ± 0.67 (subtle SD), and 3.61 ± 0.41 (obvious SD) [24]. Our results showed that computer office workers with subtle and obvious SD have significantly higher neck pain and disability. Mantana et al. reported that computer office workers with neck pain presented with tightness in the pectoralis minor, upper trapezius, and levator scapulae muscles [17]. These muscles are attached to the scapula, and their tightness may play a role in altering scapular movements, causing SD [30].

The dominant shoulder VAS scores were significantly higher in the subtle and obvious SD groups than in the normal SD group. Although the differences were not significant, the SPADI scores in the subtle and obvious SD groups tended to be higher than those in the normal SD group. However, the MCIC of SPADI is 8 points [43] and the MDC of SPADI is 18 points [44], which is greater than the difference between the normal and SD groups and the difference between each group observed in this study. Therefore, the SD investigated in subjects with observed shoulder disability and pain does not appear to be statistically or clinically different from the normal scapula. We also found that the dominant shoulder SPADI and VAS scores were higher with dominant arms in computer office workers. Alterations in scapular motion decrease the efficiency of shoulder function and can exacerbate shoulder dysfunction and pain [10]. SPADI scores showed a good correlation with NDI, neck VAS, and shoulder VAS scores in the chronic neck pain group [55]. Computer office workers reported neck and shoulder pain the most among all upper extremity complaints [56]. According to our results, NDI and neck VAS scores were significantly higher with SD; however, only the dominant shoulder VAS score was significantly higher with SD. Operating a mouse with the dominant hand may be related to SD in the dominant shoulder [57]. Efficient shoulder position, movement, stability, muscle function, and motor control are highly dependent on the function of the scapula, and scapular stability is required for the generation of muscle force [13]. The high prevalence of SD in computer office workers may be related to neck and shoulder pain and disability; however, neck pain and disability may have a greater effect on the high SD prevalence in computer office workers than shoulder pain and disability. This is consistent with a recent study examining the relationship between shoulder pain and SD that found that scapular-focused interventions had a small, clinically irrelevant effect on pain intervention in individuals with shoulder pain, and also suggests that scapular injuries commonly considered in clinical settings may not play a role in the management of patients with shoulder pain [58]. In addition, a study by Longo et al. argued that removing the cause does not rebalance the scapula, and that correcting SD does not always resolve associated shoulder pathology [59]. Therefore, further studies are necessary to investigate the relationships between neck and shoulder pain, disability, and computer office workers with SD.

Only 23% of the computer office workers were identified as having SD with LSST. There was a significant difference between the SDT and LSST results in the present study. The LSST uses a 1.5-cm distance difference between the thoracic spine and the inferior angle of the scapula bilaterally with three different shoulder positions to identify SD [26]. Depreli et al. and Ozdemir et al. reported SD prevalence rates of 41.7% and 34.1% [24,47]; however, the LSST was used to identify SD in these previous studies. Owing to the comparison and subtraction approach [26], one shoulder is assumed to have a normal SD [60]. We identified that 72.48% of the computer office workers showed subtle or obvious bilateral SD. Therefore, the LSST is not recommended to identify SD [61]. Therefore, those SD prevalence studies which used the LSST may not be suitable for detecting SD. However, the LSST is more objective than the SDT, which is an observational test [62]. The LSST is a reliable measure [62] and is recommended as a measurement of the scapular stabilizers [22]. Our results showed that the LSST position 2 distance with a subtle and obvious SD was significantly greater than the normal distance. The LSST may not be a good test to detect SD; however, it may provide reliable scale data such as distance, and a higher distance may be used as an indicator of the severity of SD. Further studies are necessary using LSST distance measurements to assess the severity of SD and improvement when the distance decreases.

Age, sex, total IT experience, working hours, and break hours were not significantly different between the normal, subtle, and obvious SD groups. Prolonged working hours using computers can lead to musculoskeletal pain [63]. However, our results showed that working hours did not differ significantly for SD. The high prevalence of neck and/or shoulder pain may have affected the prevalence of SD among the participants. Repetitive overhead arm motion and muscle fatigue increase the prevalence of SD [49,64]. However, computer office workers may not be exposed to repetitive overhead arm motion but have a high prevalence of neck pain. Further studies are needed to identify the causes of the high prevalence of SD in computer office workers.

This study had some limitations. We used the SDT to identify SD; however, there is no gold standard test for SD. Although the SDT with three grades (normal, subtle, and obvious) showed high reliability (ICC = 0.86), it is dependent upon observations [21,65]. Due to the small number of the normal SD group, further study is necessary with large numbers. Additionally, we did not specify the individual work or previous sports experience. Sex difference was also not considered. Finally, participants were recruited from Seoul, Korea. Therefore, the results may be limited to specific areas for computer office workers.

## 5. Conclusions

The prevalence of SD and neck pain among computer office workers is very high. Computer office workers with neck pain and disability showed more prevalence of SD and severe SD. The prevalence of shoulder pain was also high, but not as high as that of neck pain. Computer office workers with neck and shoulder pain had more obvious SD. Such a high rate of SD in computer office workers could be related to neck and shoulder pain and disabilities. Finally, computer office workers showed bilateral SD. Therefore, comparing the distance with the LSST bilaterally may not be suitable to identify SD; however, a higher distance could indicate the severity of SD. Further study is necessary to identify the causes of SD in computer office workers and to use the distance to grade SD.

## Figures and Tables

**Table 1:** SD with age, gender, work experience, and hours.

Scapular Dyskinesis Test	Normal Mean (95% CI)	Median (95% CI)	Subtle Mean (95% CI)	Median (95% CI)	Obvious Mean (95% CI)	Median (95% CI)	F	*p*
Age	32.5 ± 4.42 (28.08–36.92)	33 (28.0–38.0)	33.27 ± 2.1 (31.18–35.37)	33 (30.5–35.5)	31.36 ± 1.17 (30.19–32.53)	33 (30.5–33.0)	1.464	0.236
Gender (M = 1, F = 2)	1.36 ± 0.34 (1.02–1.7)	1 (1.0–1.5)	1.36 ± 0.17 (1.19–1.54)	1 (1.0–1.5)	1.43 ± 0.12 (1.31–1.55)	1 (1.5–1.5)	0.24	0.787
Total IT experience	4.23 ± 1.09 (3.14–5.31)	5.5 (3.5–5.5)	4.14 ± 0.59 (3.55–4.72)	5.5 (3.5–5.0)	4.02 ± 0.42 (3.6–4.44)	5.5 (3.5–4.5)	0.098	0.906
Working hours per day	8.59 ± 0.63 (7.96–9.23)	8.5 (8.0–9.5)	8.62 ± 0.34 (8.28–8.96)	8.5 (8.0–9.0)	8.7 ± 0.24 (8.46–8.94)	8.5 (8.5–9.0)	0.109	0.897
Working hours per week	45.82 ± 4.01 (41.81–49.82)	44 (44.0–50.0)	46.24 ± 2.04 (44.2–48.29)	44 (44.0–47.0)	45.32 ± 1.9 (43.43–47.22)	44 (44.0–47.0)	0.193	0.824
Working days per month	22.32 ± 1.22 (21.1–23.54)	21.5 (21.5–21.5)	21.91 ± 0.47 (21.44–22.37)	21.5 (21.5–21.5)	22.47 ± 0.54 (21.93–23.01)	21.5 (21.5–23.75)	0.931	0.397
Break hour per day	0.73 ± 0.22 (0.51–0.95)	0.75 (0.5–0.75)	1 ± 0.28 (0.72–1.28)	0.75 (0.75–1.125)	0.81 ± 0.13 (0.68–0.94)	0.75 (0.5–0.875)	1.361	0.261

**Table 2:** Prevalence of SD.

Scapular Dyskinesis	Normal	Subtle	Obvious
Total (*n* = 109)	11 (10.09%)	33 (30.28%)	65 (59.63%)
Dominant arm (*n* = 109)	16 (14.68%)	42 (38.53%)	51 (46.79%)
Non-dominant arm (*n* = 109)	25 (22.94%)	40 (36.70%)	44 (40.37%)

**Table 3:** SD prevalence with and without pain in the neck and shoulders.

	SDT Normal	SDT Subtle	SDT Obvious
Neck pain (*n* = 104, 95.4%)	9 (8.65%)	31 (29.81%)	64 (61.54%)
No neck pain (*n* = 5, 4.6%)	2 (40%)	2 (40%)	1 (20%)
D shoulder pain (*n* = 23, 21.1%)	2 (8.7%)	7 (30.43%)	14 (60.87%)
ND shoulder pain (*n* = 3, 2.8%)	2 (66.67%)	1 (33.34%)	0 (0%)
Both shoulder pain (*n* = 66, 60.6%)	4 (6.06%)	20 (30.30%)	42 (63.64%)
No shoulder pain (*n* = 17, 15.6%)	3 (17.65%)	5 (29.41%)	9 (52.94%)
Neck and shoulder pain (*n* = 90)	7 (7.78%)	27 (30.0%)	56 (62.22%)

D, Dominant; ND, Non-Dominant.

**Table 4:** NDI, SPADI, and VAS score with SD.

Scapular Dyskinesis Test	Normal (*n* = 11) Mean (95% CI)	Median (95% CI)	Subtle (*n* = 33) Mean (95% CI)	Median (95% CI)	Obvious (*n* = 65) Mean (95% CI)	Median (95% CI)	F	*p*	LSD
Neck NDI score	5.82 ± 3.05 (2.77–8.86)	5 (4.0–10.5)	11.76 ± 2.54 (9.21–14.3)	10 (9.5–15.0)	10.14 ± 1.36 (8.78–11.5)	9 (8.5–11)	4.094	0.019 *	Normal < Subtle Normal < Obvious
Neck VAS score	2.4 ± 1.09 (1.28–3.47)	2.4 (1.9–4.0)	3.92 ± 0.67 (3.25–4.59)	3.6 (3.5–4.8)	3.61 ± 0.41 (3.2–4.03)	3.4 (3.2–4.0)	3.29	0.041 *	Normal < Subtle Normal < Obvious
D shoulder SPADI score	4.91 ± 4.67 (0.24–9.58)	2 (4.0–15.0)	11.39 ± 3.85 (7.54–15.25)	9 (9.5–16.0)	10.72 ± 3.01 (7.72–13.73)	8 (8.0–13.0)	1.435	0.243	
ND shoulder SPADI score	2.45 ± 2.07 (0.39–4.52)	2 (2.0–7.0)	7.58 ± 3.42 (4.16–10.99)	5 (7.0–15.5)	7.06 ± 2.42 (4.64–9.49)	4 (7.0–11.5)	1.337	0.267	
D shoulder VAS score	1.52 ± 1.22 (0.3–2.74)	1.3 (1.8–4.2)	3.21 ± 0.81 (2.4–4.02)	2.7 (3.05–4.75)	2.85 ± 0.42 (2.43–3.28)	2.7 (2.85–3.7)	3.243	0.043 *	Normal < Subtle Normal < Obvious
ND shoulder VAS score	1.25 ± 0.98 (0.27–2.23)	1.2 (1.45–3.2)	2.38 ± 0.79 (1.59–3.16)	2.2 (2.9–4.5)	2.06 ±0.46 (1.6–2.52)	2.3 (2.7–3.4)	1.375	0.257	

D, Dominant; ND, Non-Dominant; NDI, Neck Disability Index; VAS, Visual Analog Scale; SPADI, Shoulder Pain and Disability Index. * *p* < 0.05.

**Table 5:** LSST length with SD.

Scapular Dyskinesis Test	Normal (*n* = 11) Mean (95% CI)	Median (95% CI)	Subtle (*n* = 33) Mean (95% CI)	Median (95%CI)	Obvious (*n* = 65) Mean (95% CI)	Median (95% CI)	F	*p*	LSD
LSST 1, mm	3.65 ± 2.33 (1.31–5.98)	2 (0.85–6.3)	5.87 ± 1.83 (4.04–7.69)	5 (3.75–6.95)	6.61 ± 1.09 (5.52–7.71)	6 (5.15–7.7)	2.042	0.135	
LSST 2, mm	2.88 ± 1.31 (1.57–4.2)	2.2 (1.3–4.05)	6.1 ± 1.49 (4.62–7.6)	5.7 (4.42–7.45)	7.38 ± 1.34 (6.03–8.72)	5.5 (5.3–8.55)	4.25	0.0168 *	Normal < Subtle Normal < Obvious
LSST 3, mm	3.14 ± 1.61 (1.53–4.74)	3 (1.3–4.9)	7.78 ± 2.26 (5.52–10.04)	6.8 (4.8–9.9)	6.98 ± 1.33 (5.66–8.31)	6.1 (5.2–7.85)	3.016	0.0532	

* *p* < 0.05.

## Data Availability

The data presented in this study are available on request from the corresponding author.
